# Association between added sugar intake and micronutrient dilution: a cross-sectional study in two adult Swedish populations

**DOI:** 10.1186/s12986-020-0428-6

**Published:** 2020-03-10

**Authors:** Esther González-Padilla, Joana A. Dias, Stina Ramne, Kjell Olsson, Cecilia Nälsén, Emily Sonestedt

**Affiliations:** 1 Nutritional Epidemiology Group, Department of Clinical Sciences Malmö, Lund University, Malmö, Sweden; 2 National Food Agency, Uppsala, Sweden

**Keywords:** Added sugar, Micronutrients, Micronutrient dilution, Nutritional recommendations, Dietary survey, Cohort

## Abstract

**Background:**

The evidence on the impact of high sugar consumption on micronutrient dilution does not yet allow for the establishment of clear thresholds of consumption. To establish upper and lower limit intake thresholds for added sugar, more studies from different countries and multiple populations are needed. The aim of this study was to examine the association between the intakes of added sugar and various micronutrients among the adult Swedish population across almost two decades.

**Methods:**

The data were obtained from the samples from two populations: 1) Riksmaten Adults, a national dietary survey (*n* = 1797, 44% male, aged 18–80 years, data collection from 2010 to 11) that assessed dietary intake using a 4-day web-based food diary; and 2) the Malmö Diet and Cancer Study, a population-based cohort study (*n* = 12,238, 45% male, aged 45–68 years, data collection from 1991 to 1994) that assessed dietary intake via a combination of a 7-day food diary, a food frequency questionnaire and an interview. The mean daily intake of nine micronutrients (calcium, folate, iron, magnesium, potassium, selenium, vitamin C, vitamin D, and zinc), adjusted for age, sex, BMI and energy intake, were examined across six added-sugar-intake groups (< 5%E, 5–7.5%E, 7.5–10%E, 10–15%E, 15–20%E, and >  20%E).

**Results:**

We observed significant inverse associations between the intake of added sugar and the intake of all micronutrients in both populations. The associations were linear; however, we could not determine the threshold of added sugar intake beyond which the micronutrient intake was clearly compromised.

**Conclusions:**

These findings suggest that in two Swedish populations the higher the intake of added sugar in the diet, the more likely it is that the intake of micronutrients will be compromised, in two Swedish populations. However, although the trends are significant and consistent with those obtained in other studies on the subject, future studies are needed in order to build the necessary scientific knowledge to establish a threshold of added sugar intake based on micronutrient dilution.

## Background

The concern about the detrimental effects that an excessive intake of added sugar (i.e., sugar added to foods during processing or preparation, not the naturally occurring sugar found in foods [1]) may have on health has grown considerably in the past few decades [2–5]. Over time, the evidence linking high added sugar consumption to the development of lifestyle-related diseases, such as obesity, cardiovascular diseases (CVDs), type 2 diabetes, and dental caries, has continued to accumulate [2, 4]. However, the recommended upper limit of added sugar intake varies in different regions and from different institutions. A systematic review identified only five guidelines that provided quantitative recommendations for sugar intake [6]. The Nordic Nutrition Recommendations (NNR) and the Dietary Guidelines for Americans suggest that added sugar should be limited to 10% of energy intake (%E) [3, 4]; however, the European Food Safety Authority (EFSA) concluded that there are insufficient scientific data to define an upper limit [7]. Moreover, a tentative recommendation to limit the intake of free sugar (i.e., added sugar plus sugar naturally occurring in honey, syrup, fruit concentrates, and fruit juices) to below 5%E has been issued by the World Health Organization (WHO) [4] and Public Health England [8]. The aforementioned systematic review has also concluded that none of the sugar guidelines meet the criteria for trustworthy recommendations and that they are based on low-quality evidence [6]. There are various arguments serving as a basis for setting the upper limit guidelines of sugar intake. For three of the guidelines mentioned in this systematic review, part of the basis considered included the increased risk of micronutrient dilution, i.e., the displacement of the intake of nutrient-dense foods by the overconsumption of energy-dense foods (rich in fat and sugar and poor in nutrients) with higher sugar intake [9, 10].

Several studies have found significant associations between the intake of added sugar and micronutrient dilution in various populations [11–15]. However, according to several reviews, the evidence is inconclusive [9, 10], mainly because of differences in the methodological approaches used in these studies. Studies in the adult population in Nordic countries are limited [16–18]. The aim of this study was, therefore, to examine whether there was an association between the intake of added sugar and the intake of micronutrients in the adult Swedish population, by examining two large population-based cohorts and their consumption patterns over almost two decades.

## Methods

### Subjects and data collection

The data used in this study were obtained from the samples from two studies: the National Swedish Food Survey of Adults (Riksmaten Adults) and the Malmö Diet and Cancer Study (MDCS). Riksmaten Adults is the most recent national dietary survey in Sweden [19], while the MDCS, although more dated and locally collected, has one of the highest-quality dietary data in the country [20, 21]. The combined data from these two studies provide information on dietary consumption for over 20 years.

In the Riksmaten Adults survey, participants were between 18 and 80 years old and resided in Sweden. The data collection process took place between May 2010 and July 2011. Based on information from the National Registry of Statistics Sweden, 5000 invitations were sent to potential participants with the intent of recruiting a representative sample of the Swedish population (in regard to gender, age group, and region). The participants completed a web-based 4-day food diary (see below) and answered a questionnaire that included questions on height and weight, as well as lifestyle and socioeconomic factors. The questionnaire was answered online, but an interviewer assisted (by telephone) those participants who could not access the website. All participants recruited for Riksmaten Adults gave oral informed consent after they received information about the study and the voluntary nature of their participation and before any measurements were performed. In total, 2268 individuals participated in Riksmaten Adults, and of the participants, 1797 (44% males) completed the food diary (36% participation rate; 31% for men and 40% for women) [19].

The MDCS is a population-based prospective cohort study in which all men born between 1923 and 1945 and women born between 1923 and 1950 who resided in Malmö (in the south of Sweden) during the data collection period (March 1991 – October 1996) were invited via a personal letter or advertisement to participate in the MDCS (*n* = 74,138) [22]. Only those with limited knowledge of Swedish or mental incapacity were excluded from participation [22]. The data collection included a dietary assessment (see below), a self-administered lifestyle and socioeconomic questionnaire, and anthropometric measurements (including height and weight). A total of 28,098 participants, of which 39% were men, were included in the study after completing the baseline survey. The participation rate was 41% (38% for men and 43% for women) [23]. In September 1994, the routine for coding dietary data was slightly altered to shorten the dietary interview from 60 to 45 min [24]. The energy intake was slightly lower after the change, and because we are investigating absolute intakes of micronutrients, we included only those individuals who completed the longer, and therefore more detailed, dietary interview (*n* = 15,107). In addition, we excluded participants who were considered to have reported an inadequate energy intake (*n* = 2869). Those energy misreporters were identified by comparing the reported energy intake with the estimated energy expenditure [25]. Thus, the final sample used in the present study included 12,238 individuals (45% men). The Ethical Committee at Lund University approved the MDCS (LU 51–90), and all the participants provided written informed consent.

### Dietary data collection

For the Riksmaten Adults survey, a web-based 4-day food diary was used to record everything that the participants had eaten or drunk during the whole day, day-by-day and meal-by-meal, as well as the time and place at which the meal was consumed. The website was linked to the national food composition database containing more than 1900 food items. If foods were missing from the survey, the participants were asked to choose the closest alternative or to register the different ingredients separately. The web tool also offered the possibility to register the method of preparation (raw, boiled, fried, etc.) to adjust for the loss of certain nutrients based on cooking methods. To cover the variation in the dietary pattern within the week, the 4-day food diary was randomly selected to start on different days of the week; the selection was divided into four rounds performed quarterly to cover seasonal variation. All the subjects who decided to take part in the study received a portion guide booklet (with pictures to help the participants estimate the portion size of the servings), a notebook (to describe the foods consumed in as much detail as possible, as well as the intake of supplements), and an information folder explaining how to register the food and navigate the food diary website. The average daily food intake was estimated based on the information from the 4-day food diary. Nutrient intake, including the intakes of monosaccharides and disaccharides (including sucrose separately), was calculated using the national food composition database [19].

For the MDCS, the dietary data were collected using a combination of three methods. First, a 7-day food diary was used to record prepared meals (lunch and dinner mostly), as well as cold drinks, and/or supplements with the intention of collecting information concerning the current diet. Second, a food frequency questionnaire was used to record the consumption frequency and portion size of 168 items that are eaten regularly and that were not covered by the food diary (covering mostly breakfast, snacks and hot drinks); portion sizes were estimated by the participants using a booklet containing pictures with 4 different portion sizes of up to 48 food items. And third, a 60-min interview with trained personnel was conducted to complete the survey; in this interview, the participants could share details regarding the method of preparation and portion sizes of the items recorded in the food diary with a trained interviewer. During the interview, the staff also checked that there was no overlap from the two sources of dietary information. The collected data were then introduced into a software program to compare the data with those from the Malmö Food and Nutrient Database, which was based on the Swedish Food Database PC KOST-93 [24, 26, 27]. The details of the MDCS data collection process [22, 23, 28] and the validity of the methods used [20, 21] are described elsewhere.

### Added sugar variable

Added sugar intake was estimated for each individual by totaling the intake of monosaccharides (mainly glucose and fructose) and sucrose from the whole diet and then subtracting the amount of monosaccharides and sucrose from fruits and berries, fruit juice, and vegetables (i.e., the main sources of naturally occurring sugars) [29]. The percentages of nonalcoholic energy intake (%E) for added sugar were calculated, and the populations were stratified into six groups according to their added sugar intake as follows: less than 5%E, 5–7.5%E, 7.5–10%E, 10–15%E, 15–20%E, and greater than 20%E from added sugar. These cut-off points were selected with the intention of comparing our results with already existing added sugar intake recommendations.

### Other dietary variables

The selection of micronutrients was performed based on the available information in the datasets, the concern expressed by the Nordic Nutrition Recommendations (NNR) regarding possible low levels in the population, and the involvement of the micronutrients in the prevention or development of lifestyle-related diseases [19], as well as their presence in previous studies on micronutrient dilution [9–18]. Ultimately, nine micronutrients were included: calcium (mg/day), folate (μg/day), iron (mg/day), magnesium (mg/day), potassium (mg/day), selenium (μg/day), vitamin C (mg/day), vitamin D (μg/day), and zinc (mg/day). The dietary composition in terms of carbohydrates, fat, protein, and fiber was calculated as the percentage of nonalcoholic energy intake.

### Statistical analysis

All statistical analyses were performed in the two populations separately using SPSS version 24 (IBM Statistics; New York, USA). Statistical significance was indicated by *p* <  0.05. Age and body mass index (BMI; calculated using height (m) and weight (kg), expressed as kg/m^2^) were analyzed for the whole sample and across the added sugar categories using an ANOVA test.

The mean daily intake and 95% confidence intervals (CIs) of the selected micronutrients and the macronutrients (as %E) were calculated for each group of added sugar intake using a general linear model adjusted for age, sex, BMI and energy intake. We also examined whether there was a linear association by using the added sugar groups as a continuous variable in the model. In addition, the number and percentage of participants with intakes below the dietary reference values (DRVs), i.e., the average requirement (AR) and recommended intake (RI) specific for the Nordic countries, as per the NNR 2012, were calculated. A chi-square test was performed to investigate whether the distribution differed between the observed and expected values. AR was defined as the nutrient level that is sufficient to cover the requirement of half of the population in a certain age and gender group, and RI is the nutrient level that meets the known requirement among almost all healthy individuals [19]. Since the DRVs are often different for men and women, sex-specific values were obtained. Recommendations for iron differ for women based on age and menopausal status. Therefore, the premenopausal threshold of iron was chosen for the female participants in Riksmaten Adults, and the postmenopausal cut-off was used for the females in the MDCS. The folate RI thresholds used for both populations were those established for individuals aged 31 and older, and the RI for vitamin D used for both populations was the value for individuals up to 74 years old.

## Results

### Population characteristics

Table 1 shows the characteristics of the two populations. The percentage of men was similar (44 and 45% in Riksmaten Adults and the MDCS, respectively), as was the mean BMI. However, the mean age was higher in the MDCS (58 years) than in Riksmaten Adults (48 years).

**Table 1:** Population characteristics

	Riksmaten Adults							Malmö Diet and Cancer Study						
	Added Sugar Intake (%E)						Total	Added Sugar Intake (%E)						Total
	< 5%	5–7.5%	7.5–10%	10–15%	15–20%	> 20%		< 5%	5–7.5%	7.5–10%	10–15%	15–20%	> 20%	
Total; N (%)	273 (15.2%)	393 (21.9%)	386 (21.5%)	521 (29.0%)	175 (9.7%)	49 (2.7%)	1797	1061 (8.7%)	2291 (18.7%)	3218 (26.3%)	424 (34.7%)	1107 (9.1%)	312 (2.5%)	12,238
Age, years; mean (SD)	48.1 (15.2)	50.2 (16.2)	50.1 (16.0)	47.1 (17.2)	43.4 (17.0)	40.6 (18.5)	48.0 (16.6)	56.8 (5.9)	57.3 (6.0)	57.6 (6.0)	57.9 (6.1)	57.6 (6.1)	58.3 (5.9)	57.6 (6.0)
BMI, kg/m^2^; mean (SD)	26.0 (4.4)	25.8 (4.4)	25.5 (4.1)	25.1 (4.3)	25.3 (4.4)	24.5 (4.1)	25.5 (4.3)	26.0 (4.2)	25.7 (3.8)	25.5 (3.7)	25.4 (3.7)	25.2 (3.7)	25.1 (4.1)	25.5 (3.8)
Males; N (%)	131 (16.5%)	172 (21.7%)	166 (21.0%)	231 (29.2%)	78 (9.8%)	14 (1.8%)	792	525 (9.6%)	1030 (18.8%)	1443 (26.4%)	1814 (33.2%)	506 (9.2%)	151 (2.8%)	5469
Age, years; mean (SD)	48.0 (16.3)	50.1 (15.9)	50.8 (16.2)	49.5 (16.8)	45.8 (16.2)	43.9 (20.4)	49.2 (16.4)	56.9 (6.0)	57.4 (6.1)	57.8 (6.0)	57.8 (6.1)	57.7 (6.2)	58.3 (5.9)	57.7 (6.1)
BMI, kg/m^2^; mean (SD)	27.0 (4.0)	26.5 (4.1)	25.8 (3.4)	25.5 (3.7)	25.8 (3.9)	25.6 (4.2)	26.0 (3.8)	26.7 (3.8)	26.2 (3.4)	26.0 (3.3)	25.8 (3.4)	25.7 (3.1)	25.6 (3.6)	26.0 (3.4)
Females; N (%)	142 (14.1%)	221 (22.0%)	220 (21.9%)	290 (28.9%)	97 (9.7%)	35 (3.4%)	1005	536 (7.9%)	1261 (18.6%)	1775 (26.2%)	2435 (36.0%)	601 (8.9%)	161 (2.4%)	6769
Age, years; mean (SD)	48.1 (14.1)	50.2 (16.5)	49.5 (15.9)	45.2 (17.3)	41.5 (17.5)	39.3 (17.9)	47.1 (16.7)	56.6 (5.9)	57.1 (5.9)	57.4 (6.1)	58.0 (6.0)	57.6 (6.0)	58.4 (5.9)	57.5 (6.0)
BMI, kg/m^2^; mean (SD)	25.1 (4.6)	25.2 (4.6)	25.3 (4.5)	24.7 (4.7)	24.9 (4.7)	24.0 (4.0)	25.0 (4.6)	25.4 (4.3)	25.3 (4.1)	25.1 (4.0)	25.0 (3.9)	24.7 (4.1)	24.6 (4.6)	25.1 (4.0)

Population characteristics for the total sample, for males and for females by added-sugar group from the Riksmaten Adults 2010–11 (*N* = 1797) and the Malmö Diet and Cancer Study (*N* = 12,238)

For the total population columns, the means and standard deviations (in brackets) are presented. For the sugar group columns, the means and standard deviations (in brackets) are presented, obtained via ANOVA are presented

*BMI* Body Mass Index, *N* Number of Observations, SD Standard Deviation

### Association between added sugar and micronutrient intakes

The mean energy intake in the MDCS (2334 kcal/day) was higher than that in Riksmaten Adults (1903 kcal/day). In addition, the mean intake of added sugar, expressed as %E, was slightly higher in the MDCS (10.1%E) than in Riksmaten Adults (9.5%E). We observed a greater proportion of participants in the group consuming less than 5% of energy from added sugar in the Riksmaten Adults than in the MDCS (15% in Riksmaten, 9% in the MDCS) and a similar frequency in the group consuming more than 20%E from added sugar (2.5%). The groups with the most participants were the groups in which participants consumed between 10 and 15%E from added sugar for both populations (29% in Riksmaten and 35% in MDCS).

For both populations, the energy intake increased as the added sugar intake increased, with approximately 250 kcal/day higher energy intakes observed in the highest added-sugar-intake group vs. the energy intakes of the lowest added-sugar-intake group. Carbohydrate intake (%E) was positively correlated with added sugar intake, whereas all other macronutrients (%E fat, %E protein, %E fiber) were negatively correlated with added sugar intake (Table 2).

**Table 2:** Energy intake and macronutrient and micronutrient intakes across the added-sugar groups

	Added Sugar Intake (%E)						
	< 5%	5–7.5%	7.5–10%	10–15%	15–20%	> 20%	p-trends
Riksmaten Adults							
N	273	393	386	521	175	49	
Energy Intake (kcal/day)	1651.3 (1578.7–1723.9)	1883.1 (1824.0–1942.3)	1899.5 (1830.6–1948.3)	2016.9 (1965.8–2068.0)	2061.1 (1971.8–2150.4)	1898.8 (1733.7–2064.0)	< 0.001
Carbohydrates (%E)	38.8 (38.0–39.6)	43.1 (42.5–43.7)	44.0 (43.4–44.6)	46.3 (45.7–46.8)	50.0 (49.1–51.0)	52.7 (51.0–54.5)	< 0.001
Fat (%E)	39.3 (38.5–40.1)	36.7 (36.1–37.3)	36.5 (35.8–37.1)	35.4 (34.8–35.9)	32.8 (31.9–33.8)	30.9 (29.1–32.6)	< 0.001
Protein (%E)	19.6 (19.2–19.9)	17.9 (17.6–18.2)	17.3 (17.0–17.6)	16.3 (16.0–16.5)	15.2 (14.8–15.7)	14.6 (13.8–15.4)	< 0.001
Fiber (%E)	2.3 (2.2–2.4)	2.3 (2.2–2.3)	2.2 (2.1–2.3)	2.1 (2.0–2.1)	1.9 (1.8–2.0)	1.8 (1.6–2.0)	< 0.001
Calcium (mg/day)	921.4 (889.7–953.1)	898.0 (877.7–923.4)	879.4 (854.2–904.6)	858.1 (836.0–880).2	838.0 (799.6–876.4)	792.1 (721.2–862.9)	< 0.001
Folate (μg/day)	284.6 (273.5–295.6)	276.6 (267.8–285.4)	266.1 (257.4–274.9)	246.1 (238.5–253.8)	232.2 (219.0–245.6)	212.6 (188.0–237.2)	< 0.001
Iron (mg/day)	10.6 (10.2–11.0)	10.7 (10.4–11.0)	10.6 (10.3–10.9)	10.3 (10.1–10.6)	9.5 (9.1–10.0)	9.6 (8.7–10.4)	< 0.001
Magnesium (mg/day)	350.9 (342.3–359.5)	343.9 (337.1–350.8)	342.6 (335.8–349.5)	322.4 (316.4–328.3)	300.1 (289.7–310.5)	289.0 (269.8–308.2)	< 0.001
Potassium (mg/day)	3298.5 (3228′7.9–3369.1)	3276.2 (3219.7–3332.7)	3175.6 (3119 .4–3231.8)	3037.2 (2988.0–3086.3)	2886.9 (2801.3–2972.5)	2604.8 (2446.9–2762.7)	< 0.001
Selenium (μg/day)	53.4 (51.3–55.4)	47.8 (46.2–49.5)	46.6 (45.0–48.2)	43.0 (41.6–44.4)	38.6 (36.2–41.1)	36.0 (31.4–40.5)	< 0.001
Vitamin C (mg/day)	97.0 (90.6–103.5)	101.2 (96.0–106.4)	96.0 (90.8–101.1)	92.3 (87.8–96.8)	94.5 (86.6–102.3)	80.4 (65.9–94.9)	0.013
Vitamin D (μg/day)	8.2 (7.7–8.8)	7.5 (7.1–7.9)	7.1 (6.6–7.5)	6.6 (6.2–7.0)	6.1 (5.4–6.8)	5.1 (3.9–6.4)	< 0.001
Zinc (mg/day)	11.7 (11.5–12.0)	11.2 (11.0–11.4)	10.8 (10.6–11.0)	10.3 (10.1–10.5)	9.6 (9.3–9.9)	9.1 (8.5–9.7)	< 0.001
Malmö Diet and Cancer Study							
N	1061	2291	3218	4249	1107	312	
Energy Intake (kcal/day)	2132.3 (2101.4–2163.1)	2238.7 (2217.8–2259.7)	2331.1 (2313.4–2348.8)	2403.6 (2388.2–2419.0)	2453.4 (2423.3–2483.6)	2403.6 (2346.9–2460.4)	< 0.001
Carbohydrates (%E)	39.9 (39.6–40.2)	41.6 (41.4–41.8)	43.3 (43.1–43.5)	45.7 (45.5–45.9)	48.8 (48.5–49.1)	52.8 (52.3–53.4)	< 0.001
Fat (%E)	40.7 (40.4–41.1)	40.2 (40.0–40.4)	39.2 (39.0–39.4)	37.7 (37.5–37.9)	35.6 (35.3–36.0)	33.0 (32.4–33.7)	< 0.001
Protein (%E)	17.3 (17.2–17.4)	16.3 (16.2–16.3)	15.6 (15.5–15.6)	14.7 (14.7–14.8)	13.8 (13.7–13.9)	12.6 (12.3–12.8)	< 0.001
Fiber (%E)	2.0 (2.0–2.0)	1.9 (1.9–2.0)	1.9 (1.9–1.9)	1.8 (1.8–1.8)	1.7 (1.7–1.7)	1.5 (1.5–1.6)	< 0.001
Calcium (mg/day)	1271.4 (1252.0–1290.8)	1207.9 (1194.7–1221.0)	1169.8 (1158.7–1180.8)	1136.5 (1126.9–1146.2)	1093.5 (1074.7–1112.4)	1012.8 (977.4–1048.3)	< 0.001
Folate (μg/day)	281.8 (278.0–285.6)	269.1 (266.5–271.6)	259.1 (257.0–261.3)	248.3 (246.0–250.2)	235.4 (231.7–239.1)	210.4 (203.4–217.3)	< 0.001
Iron (mg/day)	16.9 (16.7–17.1)	17.1 (16.9–17.2)	16.8 (16.7–16.9)	16.4 (16.3–16.5)	15.4 (15.2–15.6)	14.4 (14.0–14.7)	< 0.001
Magnesium (mg/day)	390.9 (387.6–394.1)	378.1 (375.9–380.2)	366.5 (364.7–368.3)	354.8 (353.2–356.4)	341.2 (338.1–344.4)	325.2 (319.3–331.1)	< 0.001
Potassium (mg/day)	3872.9 (3835.4–3910.3)	3687.5 (3662.1–3712.9)	3573.9 (3552.6–3595.3)	3438.2 (3419.6–3456.9)	3298.1 (3261.6–3334.6)	3053.3 (2984.8–3121.8)	< 0.001
Selenium (μg/day)	46.0 (45.3–46.7)	42.4 (41.9–42.9)	40.4 (40.0–40.8)	37.7 (37.4–38.1)	34.7 (34.1–35.4)	31.2 (29.9–32.4)	< 0.001
Vitamin C (mg/day)	118.9 (115.5–122.3)	108.0 (105.7–110.3)	105.4 (103.4–107.3)	103.8 (102.1–105.4)	108.7 (105.4–112.1)	122.8 (116.5–129.0)	0.003
Vitamin D (μg/day)	9.2 (9.0–9.3)	8.6 (8.5–8.7)	8.3 (8.2–8.4)	7.8 (7.7–7.9)	7.1 (6.9–7.2)	6.2 (5.9–6.5)	< 0.001
Zinc (mg/day)	13.1 (13.0–13.2)	12.6 (12.5–12.7)	12.1 (12.0–12.1)	11.4 (11.4–11.5)	10.6 (10.5–10.8)	9.7 (9.5–9.9)	< 0.001

Energy intake and macronutrient and micronutrient intakes (mean and 95% CI) across the added-sugar intake groups in the Riksmaten Adults (*N* = 1797) and Malmö Diet and Cancer Study (*N* = 12,238). The adjusted mean intakes and 95% confidence intervals (in brackets) are presented for the nonalcoholic energy intake (kcal/day), the macronutrient intakes, expressed as the percentage of nonalcoholic energy intake (%E) and the absolute intakes of micronutrients (mg/day or μg/day). The model was adjusted for confounders as follows: nonalcoholic energy intake was adjusted for age, sex and body mass index (BMI), macronutrient and micronutrient intakes were adjusted for age, sex, BMI and nonalcoholic energy intake

**Fig. 1:**
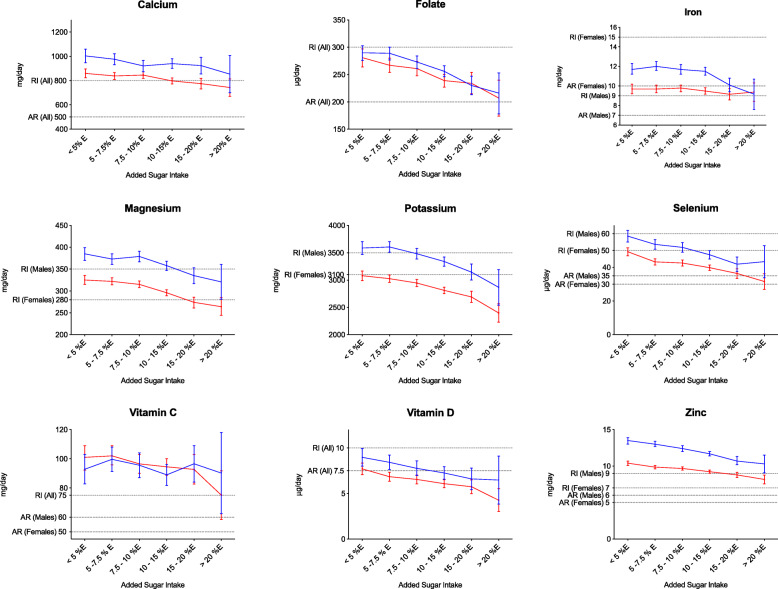
Micronutrient intake across the sugar groups for Riksmaten Adults participants. Mean intakes for men (blue) and women (red) from Riksmaten Adults are presented in relation to the dietary reference values (average requirements and recommended intake). (AR: Average Requirements; RI: Recommended Intake)

**Fig. 2:**
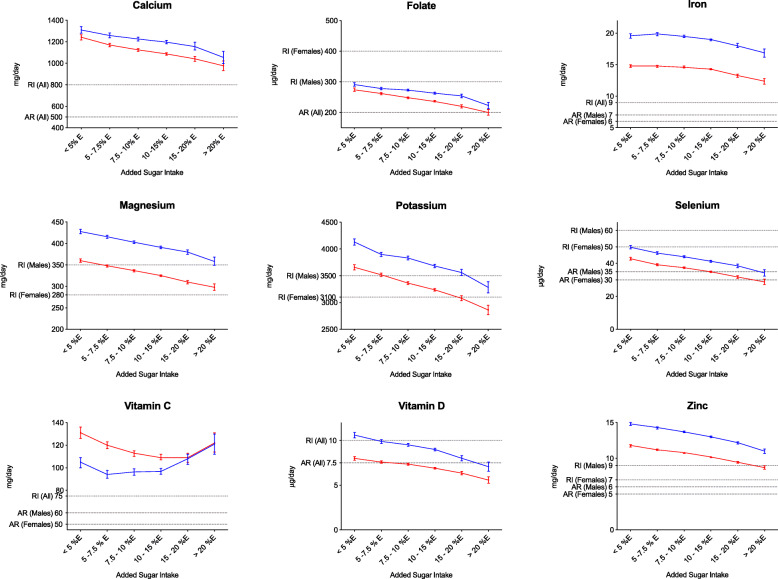
Micronutrient intake across the sugar groups for the Malmö Diet and Cancer Study participants. Mean intakes for men (blue) and women (red) from the Malmö Diet and Cancer Study are presented in relation to the dietary reference values (average requirements and recommended intake). (AR: Average Requirements; RI: Recommended Intake)

We observed an inverse association between the intake of added sugar and the daily intake of all nine micronutrients in both studies (Table 2). However, when analyzing men and women separately, iron intake in females and vitamin C intake in males showed nonsignificant linear trends in Riksmaten Adults (Additional file 1: Table S1), and vitamin C intake in males showed a significant positive trend in the MDCS (Additional file 1: Table S2). In Riksmaten Adults, the largest difference in micronutrient intake between the groups with the lowest and the highest added sugar intake was observed for vitamin D (38% decrease), selenium (33% decrease), folate (25% decrease), and zinc (22% decrease) (Fig. 1). In MDCS, the largest differences were observed for the same micronutrients, vitamin D and selenium (both decreased by 32%), zinc (26% decrease) and folate (25% decrease) (Fig. 2).

We observed that overall, micronutrient intakes in relation to the DRVs were lower in Riksmaten Adults than in the MDCS (Fig. 1 and Fig. 2). The percentage of participants from Riksmaten Adults below the AR was greatest in the group with the highest added sugar intake. For instance, the vitamin D intakes of almost 80% of the male participants and over 85% of females in this group were below the AR for vitamin D. Similarly, the percentage of participants below the AR for selenium (64% males, 43% females) and folate (50% males, 46% females) increased markedly compared to the percentage of participant below the AR in the lowest added-sugar-intake group. Furthermore, we observed similar results in the MDCS, in which more than 70% of the female participants did not meet the vitamin D AR and almost 65% of the male participants did not meet the AR for selenium (Table 3).

**Table 3:** Individuals with micronutrient intakes below dietary reference values

Riksmaten Adults									Malmö Diet and Cancer Study							
		Added Sugar Intake (%E)								Added Sugar Intake (%E)						
	DRVs^a^	< 5%	5–7.5%	7.5–10%	10–15%	15–20%	> 20%	p-trends^b^	DRVs^(1)^	< 5%	5–7.5%	7.5–10%	10–15%	15–20%	> 20%	p-trends^b^
Average Requirements (ARs)^c^																
Males																
Calcium	500 mg/day	22 (16.8%)	17 (9.9%)	14 (8.4%)	17 (7.4%)	7 (9.0%)	4 (28.6%)	0.083	500 mg/day	20 (3.8%)	16 (1.6%)	15 (1.0%)	23 (1.3%)	12 (2.4%)	10 (6.6%)	0.896
Folate	200 μg/day	41 (31.3%)	46 (26.7%)	28 (16.9%)	42 (18.2%)	21 (26.9%)	7 (50.0%)	0.112	200 μg/day	103 (19.6%)	204 (19.8%)	229 (15.9%)	335 (18.5%)	105 (20.8%)	48 (31.8%)	0.179
Iron	7 mg/day	19 (14.5%)	12 (7.0%)	10 (6.0%)	10 (4.3%)	10 (12.8%)	3 (21.4%)	0.232	7 mg/day	2 (0.4%)	2 (0.2%)	0 (0.0%)	0 (0.1%)	2 (0.4%)	0 (0.0%)	0.536
Selenium	35 μg/day	37 (28.2%)	37 (21.5%)	31 (18.7%)	60 (26.0%)	22 (28.2%)	9 (64.3%)	0.238	35 μg/day	125 (23.8%)	276 (26.8%)	405 (28.1%)	578 (31.9%)	196 (38.7%)	95 (62.9%)	< 0.001
Vitamin C	60 mg/day	59 (45.0%)	52 (30.2%)	53 (31.9%)	65 (28.1%)	25 (32.1%)	7 (50.0%)	0.050	60 mg/day	160 (30.5%)	323 (31.4%)	383 (26.5%)	458 (5.2%)	107 (21.1%)	38 (25.2%)	< 0.001
Vitamin D	7.5 μg/day	88 (67.2%)	106 (61.6%)	101 (60.8%)	140 (60.6%)	53 (67.9%)	11 (78.6%)	0.942	7.5 μg/day	152 (29.0%)	287 (27.9%)	360 (24.9%)	488 (26.9%)	193 (38.1%)	81 (53.6%)	< 0.001
Zinc	6 mg/day	8 (6.1%)	1 (0.6%)	4 (2.4%)	4 (1.7%)	1 (1.3%)	2 (14.3%)	0.316	6 mg/day	2 (0.4%)	1(0.1%)	1 (0.1%)	5 (0.3%)	4 (0.8%)	4 (2.6%)	0.003
Females																
Calcium	500 mg/day	25 (17.6%)	27 (12.2%)	24 (10.9%)	27 (9.3%)	13 (13.4%)	6 (17.1%)	0.195	500 mg/day	12 (2.2%)	20 (1.6%)	20 (1.1%)	36 (1.5%)	10 (1.7%)	9 (5.6%)	0.369
Folate	200 μg/day	47 (33.1%)	54 (24.4%)	53 (24.1%)	89 (30.7%)	41 (42.3%)	16 (45.7%)	0.019	200 μg/day	124 (23.1%)	315 (25.0%)	466 (26.3%)	718 (29.5%)	212 (35.3%)	82 (50.9%)	< 0.001
Iron	10 mg/day^(4)^	103 (72.5%)	132 (59.7%)	134 (60.9%)	177 (61.0%)	65 (67.0%)	23 (65.7%)	0.475	6 mg/day^e^	2 (0.4%)	1 (0.1%)	0 (0.0%)	2 (0.1%)	0 (0.0%)	4 (2.5%)	0.179
Selenium	30 μg/day	26 (18.3%)	46 (20.8%)	53 (24.1%)	71 (24.5%)	28 (28.9%)	15 (42.9%)	0.005	30 μg/day	113 (21.1%)	347 (27.5%)	469 (26.4%)	816 (33.5%)	263 (43.8%)	89 (55.3%)	< 0.001
Vitamin C	50 mg/day	34 (23.9%)	38 (17.2%)	39 (17.7%)	53 (18.3%)	19 (19.6%)	12 (34.3%)	0.908	50 mg/day	40 (7.5%)	109 (8.6%)	197 (11.1%)	238 (9.8%)	75 (12.5%)	23 (14.3%)	0.007
Vitamin D	7.5 μg/day	90 (63.4%)	149 (67.4%)	150 (68.2%)	209 (72.1%)	77 (79.4%)	30 (85.7%)	0.001	7.5 μg/day	259 (48.3%)	643 (51.0%)	926 (52.2%)	1369 (56.2%)	374 (62.2%)	114 (70.8%)	< 0.001
Zinc	5 mg/day	8 (5.6%)	4 (1.8%)	5 (2.3%)	10 (3.4%)	6 (6.2%)	4 (11.4%)	0.212	5 mg/day	1 (0.2%)	0 (0.0%)	1 (0.1%)	5 (0.2%)	0 (0.0%)	3 (1.9%)	0.027
Recommended Intake (RI)																
Males																
Calcium	800 mg/day	64 (48.9%)	65 (37.8%)	63 (38.0%)	78 (33.8%)	25 (32.1%)	8 (57.1%)	0.021	800 mg/day	102(19.4%)	162 (15.7%)	200 (13.9%)	235 (13.0%)	78 (15.4%)	45 (29.8%)	0.301
Folate	300 μg/day	89 (67.9%)	120 (69.8%)	112 (67.5%)	155 (67.1%)	64 (82.1%)	13 (92.9%)	0.144	300 μg/day	361 (68.8%)	697 (67.7%)	975 (67.6%)	1269 (70.0%)	373 (73.7%)	134 (88.7%)	< 0.001
Iron	9 mg/day	49 (37.4%)	49 (28.5%)	33 (19.9%)	49 (21.2%)	29 (37.2%)	10 (71.4%)	0.429	9 mg/day	4 (0.8%)	10 (1.0%)	9 (0.6%)	6 (0.3%)	6 (1.2%)	4 (2.6%)	0.942
Magnesium	350 mg/day	75 (57.3%)	85 (49.4%)	70 (42.2%)	97 (42.0%)	35 (44.9%)	11 (78.6%)	0.050	350 mg/day	180 (34.3%)	300 (29.1%)	423 (29.3%)	542 (29.9%)	165 (32.6%)	70 (46.4%)	0.266
Potassium	3500 mg/day	89 (67.9%)	98 (57.0%)	85 (51.2%)	117 (50.6%)	45 (57.7%)	14 (100%)	0.117	3500 mg/day	199 (37.9%)	400 (38.8%)	551 (38.2%)	756 (41.7%)	232 (45.8%)	91 (60.3%)	< 0.001
Selenium	60 μg/day	94 (71.8%)	123 (71.5%)	123 (74.1%)	179 (77.5%)	62 (79.5%)	11 (78.6%)	0.068	60 μg/day	431 (82.1%)	861 (83.6%)	1264 (87.6%)	1650 (91.0%)	472 (93.3%)	144 (95.4%)	< 0.001
Vitamin C	75 mg/day	71 (54.2%)	73 (42.4%)	74 (44.6%)	98 (42.4%)	38 (48.7%)	8 (57.1%)	0.396	75 mg/day	225 (42.9%)	481 (46.7%)	580 (40.2%)	718 (39.6%)	158 (31.2%)	49 (32.5%)	< 0.001
Vitamin D	10 μg/day^f^	97 (74.0%)	127 (73.8%)	136 (81.9%)	173 (74.9%)	63 (80.8%)	12 (85.7%)	0.302	10 μg/day^f^	323 (61.5%)	639 (62.0%)	885 (61.3%)	1170 (64.5%)	363 (71.7%)	127 (84.1%)	< 0.001
Zinc	9 mg/day	28 (21.4%)	23 (13.4%)	24 (14.5%)	34 (14.7%)	13 (16.7%)	5 (35.7%)	0.729	9 mg/day	41 (7.8%)	67 (6.5%)	79 (5.5%)	112 (6.2%)	42 (8.3%)	36 (23.8%)	0.010
Females																
Calcium	800 mg/day	88 (62.0%)	103 (46.6%)	109 (49.5%)	145 (50.0%)	47 (48.5%)	17 (48.6%)	0.160	800 mg/day	101 (18.8%)	230 (18.2%)	304 (17.1%)	439 (18.0%)	123 (20.5%)	44 (27.3%)	0.161
Folate	300 μg/day^g^	109 (76.8%)	159 (71.9%)	166 (75.5%)	228 (78.6%)	83 (85.6%)	30 (85.7%)	0.016	300 μg/day^g^	385 (71.8%)	977 (77.5%)	1424 (80.2%)	2014 (82.7%)	519 (86.4%)	145 (90.1%)	< 0.001
Iron	15 mg/day^d^	139 (97.9%)	209 (94.6%)	201 (91.4%)	269 (92.8%)	91 (93.8%)	31 (88.6%)	0.050	9 mg/day^e^	37 (6.9%)	55 (4.4%)	47 (2.6%)	84 (3.4%)	34 (5.7%)	18 (11.2%)	0.827
Magnesium	280 mg/day	78 (54.9%)	81 (36.7%)	84 (38.2%)	112 (38.6%)	51 (52.6%)	20 (57.1%)	0.989	280 mg/day	118 (22.0%)	290 (23.0%)	376 (21.2%)	574 (23.6%)	173 (28.8%)	57 (35.4%)	0.001
Potassium	3100 mg/day	99 (69.7%)	126 (57.0%)	137 (62.3%)	176 (60.7%)	66 (68.0%)	28 (80.0%)	0.539	3100 mg/day	186 (34.7%)	473 (37.5%)	698 (39.3%)	1067 (43.8%)	303 (50.4%)	105 (65.2%)	< 0.001
Selenium	50 μg/day	92 (64.8%)	155 (70.1%)	158 (71.8%)	232 (80.0%)	81 (83.5%)	31 (88.6%)	< 0.001	50 μg/day	424 (79.1%)	1066 (84.5%)	1547 (87.2%)	2201 (90.4%)	558 (92.8%)	154 (95.7%)	< 0.001
Vitamin C	75 mg/day	69 (48.6%)	78 (35.3%)	92 (41.8%)	119 (41.0%)	43 (44.3%)	19 (54.3%)	0.639	75 mg/day	135 (25.2%)	319 (25.3%)	517 (29.1%)	709 (29.1%)	178 (29.6%)	48 (29.8%)	0.008
Vitamin D	10 μg/day^f^	109 (76.8%)	177 (80.1%)	184 (83.6%)	247 (85.2%)	87 (89.7%)	35 (100%)	< 0.001	10 μg/day^f^	442 (82.5%)	1071 (84.9%)	1524 (85.9%)	2114 (86.8%)	537 (89.4%)	150 (93.2%)	< 0.001
Zinc	7 mg/day	25 (17.6%)	34 (15.4%)	30 (13.6%)	43 (14.8%)	23 (23.7%)	11 (31.4%)	0.177	7 mg/day	24 (4.5%)	42 (3.3%)	62 (3.5%)	141 (5.8%)	46 (7.7%)	34 (21.1%)	< 0.001

Individuals below the average requirement and recommended intake by added-sugar group from Riksmaten Adults (*N* = 1797) and the Malmö Diet and Cancer Study (*N* = 12,238)

The frequencies and percentages (in brackets) of individuals with micronutrient intakes below the Dietary Reference Values (DRVs) according to the Nordic Nutrition Recommendations 2012 are presented by added sugar intake groups

*DRVs* Dietary Reference Values

^a^ DRVs for a Nordic population according to NNR 2012 for adult males and females. ^b^ Statistical significance for the trends was established at *p* < 0.050. ^c^ No AR values for magnesium or potassium. ^d^ Pre-menopausal value. ^e^ Post-menopausal value. ^f^ Recommendation for adults up to 74 years old. ^g^ Recommendation for adults aged 31 and older

## Discussion

We observed significant negative associations between the intake of added sugar and the intake of micronutrients in two Swedish populations of adults. However, we were not able to ascertain a clear threshold of added sugar intake beyond which the decrease in micronutrient intake was remarkably enlarged.

In line with our study, previous studies around the world have found significant associations between the intake of added sugar and micronutrient dilution regardless of differences in methodologies and populations [11–18]. In older adults in Australia, those with added sugar intakes above 10%E were more likely to have poor micronutrient intakes [15]. Similarly, in Australian children and adolescents, higher intakes of added sugars were associated with intakes of nutrient-poor (energy-dense) foods [14]. In South Africa, a study with older adults of mixed ancestry showed evidence of micronutrient dilution in both men and women with increasing sugar intake [13] and in elderly black women; their overall poor diet quality was partly explained by the displacement of micronutrients by added sugars in the diet [12]. In the United States, Bowman found that an intake of 18%E or more from added sugars in a population aged 2 years and older was associated with the lowest intakes of all measured micronutrients [11]. This phenomenon has also been studied in a few Nordic populations, such as with children in Denmark, where a clear trend of declining micronutrient density was observed with increasing added sugar intake [16]. In Norway, a study of children at different stages of school years showed a negative association between added sugar intake and intakes of fiber and several micronutrients; in many cases, those in the highest quartile did not reach the micronutrient recommendations [17]. The only study based on a Swedish population was published in 1983; the authors examined sucrose intake (the main sugar added to foods in Sweden) of adults over the age of 20 who had participated in a dietary survey carried out between 1973 and 1976. The participants were classified into three groups: “low sugar consumption” (lowest decile), “high sugar consumption” (highest decile), and “normal sugar consumption” (the remaining participants). This study revealed no decreases in nutrient intakes compared to the DRVs regardless of the sugar-intake group; the exception was iron intake, which was low for women in both the high and low sugar consumption groups. The aforementioned study did not look for trends across sugar-intake groups [18]. Some reviews [9, 10] have concluded that the evidence regarding this topic is inconclusive due to the differences in methodological approaches and the lack of consensus on the type of sugar measured and the micronutrients considered. However, despite the differences in the populations and micronutrients selected, as well as differences in certain methodological aspects, the overall result of all these studies (including our own) was an inverse association between sugar intake and micronutrient intake, suggesting the existence of micronutrient dilution. Some of the aforementioned studies expressed their results in terms of tertiles [12, 13], quartiles [17] or quintiles [14, 16] of added sugar intake, which makes it difficult to compare the results for the predefined intake groups found in our study to those of other studies.

Although several studies have already investigated the association between added sugar and micronutrient dilution, it is important to conduct analyses in different populations because of different food habits. In addition, in some countries, sugary foods can be fortified with micronutrients, which might mask the association between added sugar intake and micronutrient dilution [30]. In Sweden, unhealthy foods are not commonly fortified, and therefore, there might be an even stronger association in our population. A major strength of our study is that we used the same added sugar definition and the same nine micronutrients in two Swedish populations during different time periods, indicating a consistent relationship between added sugar and micronutrient dilution in the Swedish population.

The DRVs (ARs and RIs) used to establish adequate micronutrient intake levels were drawn from the NNR 2012 [3], a nutritional recommendation designed specifically for Nordic populations and Nordic dietary patterns. The use of these DRVs is also a strength of this study since the variation in diet between populations may lead to different recommendations for the intake of micronutrients based on food availability, food culture, and dietary preferences within the population [10].

Nonalcoholic energy intake was used based on our goal to explore the dietary composition associated with added sugar intake. Alcoholic beverages are highly caloric and only minimally contribute to micronutrient or sugar intake. Including the energy derived from alcoholic beverages in our analysis could have potentially skewed our results; a high intake of alcoholic beverages could appear as a lower %E coming from added sugar compared to when only nonalcoholic foods and beverages are included.

The sample size was rather limited in the Riksmaten Adults survey (*n* = 1797), although the data collection method was performed in a way that aimed to obtain a sample that was representative of the national population. The sample size for the MDCS was rather large, but it included participants only from the city of Malmö, in southern Sweden. The participation rates were only 36% in the Riksmaten Adults (for the food diary) and 40% in the MDCS. Those who agreed to participate in Riksmaten Adults had a higher level of education and a slightly higher annual income than those who declined to participate [19], while participants and nonparticipants in the MDCS had a similar socioeconomic status. Nonparticipants in both Riksmaten Adults and the MDCS were more likely to have been born outside of Sweden [19, 22, 23]. However, while the data pertaining to the MDCS are quite dated (the data collection took place during the 1990s), the data collected for Riksmaten Adults is the most recent available data sampling the total Swedish adult population (2010–2011). The age difference between the populations should be noted. While Riksmaten Adults covered the entirety of the adult population from age 18 to 80 years old, the MDCS focused solely on older adults (ranging 45–68); therefore, the results of this study might not fully represent the population outside the 45–68 age range since the sample of individuals outside that age range was much smaller. This study, however, fairly accurately reflected the dietary patterns of the Swedish population over the past two decades [19, 22, 23]. Despite the apparent differences between the two populations, the results obtained were similar for both samples, indicating a tendency that has been perpetuated for almost 20 years.

The dietary data were self-reported in both populations, which constitutes a challenge in nutritional epidemiology studies. Misreporting is a well-known phenomenon within nutritional epidemiology and diet-related research [31] that we should, whenever possible, attempt to account for in the analysis. Commonly, individuals tend to underreport less healthy foods and overreport healthier foods [25]. In our study, we had information regarding potential energy misreporting for the participants in the MDCS; however, such information was not available for the participants in Riksmaten Adults. In Riksmaten Adults, a 4-day food diary was used; in the MDCS, a combination of a food diary and a food frequency questionnaire (FFQ) was used. One of the advantages of food diaries, as used in both populations (4-day web-based for Riksmaten Adults and 7-day food diary for the MDCS), is the diminished risk for recall bias as participants register their food intake on a meal-by-meal basis [19]. The FFQ used in the MDCS contained detailed questions regarding sugary foods, and the 7-day food diary asked about the consumption of sugar-sweetened beverages. Thus, the validity of sugar intake was high compared to the reference method (correlation coefficients for sucrose with the reference method: 0.60 for men and 0.74 for women) [21]. Overall, the validity of the dietary methods used in both studies (published elsewhere for both Riksmaten Adults [32] and the MDCS [21, 33]) appears to be relatively high.

## Conclusions

In conclusion, the observed inverse association between the intake of added sugar and the intake of micronutrients in the two populations studied supports the occurrence of micronutrient dilution. Ultimately, these findings suggest that the higher the intake of added sugar in the diet, the more likely it is that the intake of micronutrients will be compromised.

These results complement previous literature in the field, supporting the claim that higher levels of added sugar intake may be associated with a lower intake of micronutrients. Although a significant negative trend was clear and consistent for all micronutrients studied, no specific threshold could be established to fit all the micronutrients under consideration to provide an evidence-based recommendation for added sugar intake. Future studies are needed to clarify whether there is a threshold effect between added sugar intake and micronutrient dilution.

## Supplementary information

**Additional file 1: Table S1.** Micronutrient and energy intakes by sex across the added-sugar groups in Riksmaten Adults. **Table S2.** Micronutrient and energy intakes by sex across the added-sugar groups in the Malmö Diet and Cancer Study.

## Data Availability

MDCS: The dataset used and analyzed during the current study is available from the corresponding author on reasonable request. Riksmaten Adults: The data that support the findings of this study are available from Livsmedelsverket (Swedish National Food Agency) but restrictions apply to the availability of these data, which were used under license for the current study and are not publicly available. However, the data are available from the authors upon reasonable request and with the permission of Livsmedelsverket (Swedish National Food Agency).

## Abbreviations

%E: Percentage of nonalcoholic energy intake
AR: Average Requirement
BMI: Body Mass Index
CI: Confidence Interval
CVD: Cardiovascular Disease
DRV: Dietary Reference Value
EFSA: European Food Safety Authority
FFQ: Food Frequency Questionnaire
kg: Kilograms
m: Meters
MDCS: Malmö Diet and Cancer Study
mg/day: Milligrams per day
NNR: Nordic Nutrition Recommendations
RI: Recommended Intake
WHO: World Health Organization
μg/day: Micrograms per day
