# Supplementary material for: Association between added sugar intake and micronutrient dilution: a cross-sectional study in two adult Swedish populations
Source: Nutr Metab (Lond). 2020 Mar 10;17:15. doi: 10.1186/s12986-020-0428-6 (PMC7011604; doi:10.1186/s12986-020-0428-6)
Supplement: Supplementary file 1 — Additional file 1: Table S1. Micronutrient and energy intakes by sex across the added-sugar groups in Riksmaten Adults. Table S2. Micronutrient and energy intakes by sex across the added-sugar groups in the Malmö Diet and Cancer Study. [file 12986_2020_428_MOESM1_ESM.docx]

**Supplementary Tables:**

**Table S1. Micronutrient and energy intakes by sex the across added-sugar groups in Riksmaten Adults.**

|  |  | **Added Sugar Intake (%E)** | | | | | |  |
| --- | --- | --- | --- | --- | --- | --- | --- | --- |
|  |  | **< 5%** | **5 – 7.5%** | **7.5 –10%** | **10 – 15%** | **15 – 20%** | **> 20%** | **p-trends** |
| **ENERGY INTAKE** | | | | | | | | |
|  | Males (kcal/day) | 1845.7  (173.21 – 1960.1) | 2124.5  (2028.2 – 2220.7) | 2147.1  (2049.6 – 2244.7) | 2267.4  (2185.1 – 2349.7) | 2359.8  (2216.0 – 2503.6) | 1972.3  (1651.0 – 2293.6) | < 0.001 |
|  | Females (kcal/day) | 1493.7  (1414.3 – 1573.1) | 1706.3  (1643.3 – 1769.4) | 1709.2  (1647.4 – 1771.1) | 1817.6  (1762.6 – 1872.5) | 1825.7  (1729.9- 1921.6) | 1862.4  (1703.5 – 2021.3) | < 0.001 |
| **MALES** | | | | | | | | |
|  | Calcium (mg/day) | 1003.3  (947.9 – 1058.7) | 976.6  (930.9 – 1022.3) | 920.8  (874.5 – 967.1) | 940.0  (900.6 – 979.3) | 923.7  (855.0 – 992.4) | 853.2  (700 .5- 1005.9) | 0.013 |
|  | Folate (μg/day) | 289.7  (276.0 – 303.4) | 288.1  (277.5 – 300.1) | 273.0  (261.6 – 284.5) | 256.1  (246.4 – 265.9) | 230.4  (213.4 – 247.4) | 215.7  (177.9 – 253.6) | < 0.001 |
|  | Iron (mg/day) | 11.7  (11.2 - 12.3) | 12.0  (11.6 - 12.5) | 11.7  (11.2 - 12.2) | 11.5  (11.1 - 11.9) | 10.1  (9.4 - 10.8) | 9.1  (7.6 - 10.7) | < 0.001 |
|  | Magnesium (mg/day) | 384.7  (370.2 – 399.1) | 373.1  (361.1 – 385.0) | 378.9  (366.8 – 390.9) | 357.6  (347.4 – 367.9) | 335.3  (317.4 – 353.2) | 320.9  (281.1 – 360.7) | < 0.001 |
|  | Potassium (mg/day) | 3588.1  (3470.6 – 3705.7) | 3608.0  (3511.0 – 3704.9) | 3483.0  (3384.8 – 3581.3) | 3339.6  (3256 .2- 3423.0) | 3147.9  (3002.2 – 3293.6) | 2866.7  (2542.7 – 3190.6) | < 0.001 |
|  | Selenium (μg/day) | 58.6  (55.1 - 62.0) | 53.7  (50.8 - 56.5) | 51.9  (49.0 - 54.7) | 47.5  (45.0 - 49.9) | 41.9  (37.6 - 46.2) | 43.4  (33.9 - 52.9) | < 0.001 |
|  | Vitamin C (mg/day) | 92.9  (82.8 – 103.0) | 99.7  (91.4 – 108.0) | 95.6  (87.1 – 104.0) | 89.0  (81.8 - 96.2) | 96.6  (84.1 – 109.1) | 90.3  (62.4- 118.1) | 0.416 |
|  | Vitamin D (μg/day) | 9.0  (8.0 - 9.9) | 8.4  (7.6 - 9.2) | 7.8  (7.0 - 8.6) | 7.2  (6.6 - 7.9) | 6.6  (5.4 - 7.8) | 6.5  (3.8 - 9.1) | < 0.001 |
|  | Zinc (mg/day) | 13.5  (13.0 - 13.9) | 13.0  (12.7 - 13.4) | 12.4  (12.0 - 12.8) | 11.7  (11.4 - 12.0) | 10.7  (10.2 - 11.3) | 10.3  (9.1 - 11.5) | < 0.001 |
| **FEMALES** | | | | | | | | |
|  | Calcium (mg/day) | 859.3  (823.1 – 895.6) | 838.3  (810.0 – 866.6) | 845.9  (818.2 – 873.6) | 796.9  (772.1 – 821.6) | 775.2  (732.1 – 818.3) | 742.7  (671.3 – 814.1) | < 0.001 |
|  | Folate (μg/day) | 281.0  (264.4 – 297.5) | 267.2  (254.3 – 280.1) | 261.1  (248.5 – 273.8) | 238.6  (227.3 – 249.9) | 234.6  (214.9 – 254.2) | 207.1  (174.6 – 239.6) | < 0.001 |
|  | Iron (mg/day) | 9.7  (9.2 - 10.2) | 9.7  (9.3 - 10.1) | 9.8  (9.4 - 10.1) | 9.5  (9.1 - 9.8) | 9.1  (8.6 - 9.7) | 9.4  (8.4 - 10.3) | 0.121 |
|  | Magnesium (mg/day) | 325.4  (315.0 – 335.7) | 321.9  (313.8- 330.0) | 315.6  (307.6 – 323.5) | 296.0  (288.9 – 303.1) | 273.8  (261.5 – 286.1) | 264.3  (243.9 – 284.7) | < 0.001 |
|  | Potassium (mg/day) | 3079.9  (2993.4 - 3166.5) | 3025.4  (2957.9- 3093.0) | 2944.8  (2878.6 – 3011.0) | 2811.8  (2752.6 – 2871.0) | 2693.2  (2590.3 – 2796.1) | 2399.1  (22298.7- 2569.5) | < 0.001 |
|  | Selenium (μg/day) | 49.3  (46.9 - 51.6) | 43.3  (41.4 - 45.1) | 42.6  (40.8 - 44.4) | 39.8  (38.2 - 41.4) | 36.4  (33.5 - 39.2) | 31.6  (26.9 - 36.2) | < 0.001 |
|  | Vitamin C (mg/day) | 100.8  (92.3 – 109.3) | 102.5  (95.9 – 109.2) | 96.5  (90.1 – 103.0) | 94.5  (88.7 – 100.3) | 92.8  (82.7 – 102.8) | 75.1  (58.4 - 91.8) | 0.005 |
|  | Vitamin D (μg/day) | 7.7  (7.1 - 8.3) | 6.8  (6.3 - 7.3) | 6.5  (6.1 - 7.0) | 6.1  (5.6 - 6.5) | 5.7  (5.0 - 6.5) | 4.3  (3.0 - 5.5) | < 0.001 |
|  | Zinc (mg/day) | 10.4  (10.1 - 10.7) | 9.8  (9.6 - 10.1) | 9.7  (9.4 - 9.9) | 9.2  (9.0 - 9.4) | 8.8  (8.4 - 9.1) | 8.2  (7.6 - 8.8) | < 0.001 |

**Micronutrient and energy intakes by sex (mean and 95% CI) across the added-sugar intake in Riksmaten Adults (N = 1797).**

The adjusted mean intakes and 95% confidence intervals (in brackets) are presented for the nonalcoholic energy intake (kcal/day), the macronutrient intakes, expressed as the percentage of nonalcoholic energy intake (%E) and the absolute intakes of micronutrients (mg/day or μg/day). The model was created via a general linear model and adjusted for confounders as follows: nonalcoholic energy intake was adjusted for age and body mass index (BMI), and macronutrient and micronutrient intakes were adjusted for age, BMI and nonalcoholic energy intake

**Table S2. Micronutrient and energy intakes by sex across the added-sugar groups in the Malmö Diet and Cancer Study.**

|  |  | **Added Sugar Intake (E%)** | | | | | |  |
| --- | --- | --- | --- | --- | --- | --- | --- | --- |
|  |  | **< 5%** | **5 – 7.5%** | **7.5 –10%** | **10 – 15%** | **15 – 20%** | **> 20%** | **p-trends** |
| **ENERGY INTAKE** | | | | | | | | |
|  | Males (kcal/day) | 2387.5  (2336.1 – 2438.9) | 2549.1  (2512.5 – 2585.7) | 2634.9  (2604.0 – 2665.8) | 2719.3  (2691.7 – 2746.8) | 2767.9  (2715.7 – 2820.1) | 2673.4  (2577.9 – 2768.8) | < 0.001 |
|  | Females (kcal/day) | 1936.3  (1900.2 – 1972.5) | 1989.2  (1965.6 – 2012.7) | 2087.0  (2067.2 – 2106.9) | 2147.5  (2130.6 – 2164.5) | 2198.8  (2164.6 – 2232.9) | 2189.2  (2123.3 – 2255.1) | < 0.001 |
| **MALES** | | | | | | | | |
|  | Calcium (mg/day) | 1310.2  (1279.6 – 1340.9) | 1258.9  (1237.2 – 1280.6) | 1225.7  (1207.4 – 1244.0) | 1197.0  (1180.6 – 1213.3) | 1155.2  (1124.2 – 1186.1) | 1054.8  (998.3 – 1111.3) | < 0.001 |
|  | Folate (μg/day) | 291.5  (285.9 – 297.2) | 278.4  (274.4 – 2825) | 273.1  (269.8 – 276.5) | 263.0  (260.0 – 266.0) | 254.2  (248.4 – 259.9) | 222.9  (212.4 – 233.3) | < 0.001 |
|  | Iron (mg/day) | 19.6  (19.2 – 19.9) | 19.9  (19.6 – 20.1) | 19.5  (19.3 – 19.7) | 19.0  (18.8 – 19.1) | 18.0  (17.7 – 18.4) | 16.9  (16.2 – 17.5) | < 0.001 |
|  | Magnesium (mg/day) | 428.3  (423.2 – 433.3) | 415.9  (412.4 – 419.5) | 403.3  (400.4 – 406.3) | 391.1  (388.4 – 393.7) | 379.9  (374.8 – 384.9) | 358.4  (349.2 – 367.7) | < 0.001 |
|  | Potassium (mg/day) | 4131.3  (4074.0 – 4188.7) | 3900.4  (3859.8 – 3941.1) | 3833.2  (3799.0 – 3867.4) | 3684.4  (36543.8– 3715.0) | 3563.3  (3505 4 – 3621.2) | 3285.8  (3180.1 – 3391.5) | < 0.001 |
|  | Selenium (μg/day) | 49.8  (48.7 – 50.9) | 46.3  (45.6 – 47.1) | 44.0  (43.4 – 44.7) | 41.3  (40.7 – 41.9) | 38.5  (37.4 – 39.6) | 34.2  (32.3 – 36.2) | < 0.001 |
|  | Vitamin C (mg/day) | 104.7  (99.9 – 109.5) | 94.1  (90.7 – 97.6) | 96.3  (93.4 – 99.2) | 96.8  (94.3 – 99.4) | 107.7  (102.8 – 112.5) | 121.1  (112.2 – 130.1) | < 0.001 |
|  | Vitamin D (μg/day) | 10.6  (10.3 – 10.9) | 9.9  (9.7 – 10.1) | 9.5  (9.3 – 9.7) | 9.0  (8.8 – 9.1) | 8.0  (7.7 - 8.3) | 7.1  (6.5 – 7.6) | < 0.001 |
|  | Zinc (mg/day) | 14.8  (14.6 – 15.0) | 14.3  (14.1 – 14.4) | 13.7  (13.6 – 13.8) | 13.0  (12.9 – 13.1) | 12.2  (12.0 – 12.3) | 11.0  (10.7 – 11.3) | < 0.001 |
| **FEMALES** | | | | | | | | |
|  | Calcium (mg/day) | 1241.8  (1217.4 – 1266.2) | 1169.7  (1153.8 – 1185.7) | 1124.8  (1111.4 – 1138.1) | 1087.4  (1075.9 – 1098.8) | 1041.5  (1018.5 – 1064.5) | 976.7  (932.4 – 1020.1) | < 0.001 |
|  | Folate (μg/day) | 274.5  (269.4 – 279.6) | 261.9  (258.5 – 265.2) | 247.9  (245.1 – 250.6) | 236.4  (234.0 – 238.8) | 219.9  (215.0 – 224.7) | 199.9  (190.6 – 209.2) | < 0.001 |
|  | Iron (mg/day) | 14.8  (14.6 – 15.0) | 14.8  (14.6 – 14.9) | 14.6  (14.5 – 14.8) | 14.3  (14.2 – 14.4) | 13.3  (13.0 – 13.5) | 12.4  (11.9 – 12.8) | < 0.001 |
|  | Magnesium (mg/day) | 360.2  (356.1 – 364.4) | 347.7  (345.0 – 350.4) | 336.8  (334.5 – 339.1) | 325.4  (323.5 – 327.4) | 309.8  (305.9 – 313.7) | 298.5  (291.0 – 306.1) | < 0.001 |
|  | Potassium (mg/day) | 3659.0  (3609.9 – 3708.1) | 3520.2  (3488 .2– 3552.1) | 3364.7  (3338.2 – 3391.4) | 3238.7  (3215.7 – 3261.7) | 3080.4  (3034.1 – 3126.6) | 2861.8  (2772.8 – 2950.7) | < 0.001 |
|  | Selenium (μg/day) | 42.8  (42.0 – 43.7) | 39.2  (38.6 – 39.8) | 37.4  (36.9 – 37.9) | 34.9  (34.5 – 35.3) | 31.7  (30.8 – 32.5) | 28.8  (27.2 – 30.4) | < 0.001 |
|  | Vitamin C (mg/day) | 131.1  (126.3 – 135.9) | 119.7  (116.6 – 122.8) | 112.7  (110.1 – 115.3) | 109.4  (107.1 – 111.6) | 109.4  (104.5 – 113.5) | 122.5  (1143.8– 131.2) | < 0.001 |
|  | Vitamin D (μg/day) | 8.0  (7.8 – 8.2) | 7.6  (7.4 – 7.7) | 7.3  (7.2 – 7.4) | 6.9  (6.8 – 7.0) | 6.3  (6.1 – 6.5) | 5.6  (5.2 – 5.9) | < 0.001 |
|  | Zinc (mg/day) | 11.8  (11.6 – 11.9) | 11.2  (11.1 – 11.3) | 10.8  (10.7 – 10.8) | 10.2  (10.1 – 10.2) | 9.4  (9.3 – 9.6) | 8.7  (8.5 – 8.9) | < 0.001 |

**Micronutrient and energy intakes by sex (mean and 95% CI) across the added-sugar intake groups in the Malmö Diet and Cancer Study (N = 12238).**

The adjusted mean intakes and 95% confidence intervals (in brackets) are presented for the nonalcoholic energy intake (kcal/day), the macronutrient intakes, expressed as the percentage of nonalcoholic energy intake (%E) and the absolute intakes of micronutrients (mg/day or μg/day). The model was created via a general linear model and adjusted for confounders as follows: nonalcoholic energy intake was adjusted for age and body mass index (BMI), and macronutrient and micronutrient intakes were adjusted for age, BMI and nonalcoholic energy intake.
